# Draft Genome Sequences of Two Toxigenic *Corynebacterium ulcerans* Strains

**DOI:** 10.1128/genomeA.00699-15

**Published:** 2015-06-25

**Authors:** Marc-Christian Domingo, Eric Fournier, Cynthia Massé, Hugues Charest, Kathryn Bernard, Jean-Charles Côté, Cécile Tremblay

**Affiliations:** a Laboratoire de Santé Publique du Québec, Institut National de Santé Publique du Québec, Sainte-Anne-de-Bellevue, QC, Canada; b Département de Microbiologie-Infectiologie et d'Immunologie, Université Laval, Québec, QC, Canada; c Département de Microbiologie, Infectiologie et Immunologie, Université de Montréal, Montréal, QC, Canada; d Public Health Agency of Canada, National Microbiology Laboratory, Winnipeg, MB, Canada

## Abstract

Here, we present the draft genome sequences of two toxigenic *Corynebacterium ulcerans* strains isolated from two different patients: one from a blood sample and the other from a scar exudate following surgery. Although these two strains harbor the diphtheria toxin gene *tox*, no full prophage sequences were found in the flanking regions.

## GENOME ANNOUNCEMENT

Three *Corynebacterium* species are capable of producing the diphtheria toxin: *C. diphtheriae*, *C. ulcerans* and, to a lesser extent, *C. pseudotuberculosis*. The toxin is encoded by the *tox* gene located within a prophage lysogenized in the bacterial chromosome (1, 2). In developing countries, diphtheria is caused by toxigenic *C. diphtheriae*. In developed countries, however, diphtheria-like infections caused by toxigenic *C. ulcerans* have outnumbered those caused by toxigenic *C. diphtheriae* (3, 4).

Here, we present the draft genome sequences of two toxigenic *C. ulcerans* strains. Strains LSPQ-04227 (NML 040264) and LSPQ-04228 (NML 130346) were isolated from two different patients, one from a blood sample and the other from a scar exudate following thoracic surgery. Both strains were identified as *C. ulcerans* based on growth on Tinsdale plates (5), biochemical profiles on bioMérieux API Coryne microbial identification strips, and 16S rRNA gene sequences. The presence of the *tox* gene, typical of toxigenic strains, was confirmed by PCR (6). An Elek’s test (7) confirmed the toxigenicity of both strains.

Both genomes were sequenced on an Ion Torrent PGM sequencer (Life Technologies, Carlsbad, CA). A total of 4,230,671 reads were sequenced for a total of 653 Mbp for isolate LSPQ-04227, while 2,672,954 reads were sequenced for a total of 468 Mbp for isolate LSPQ-04228. The quality of the raw sequence data was checked using FastQC (http://www.bioinformatics.babraham.ac.uk/projects/fastqc/). SPAdes version 3.5 (http://bioinf.spbau.ru/spades [8]) was used to assemble each genome. The annotations were done using the NCBI Prokaryotic Genome Annotation Pipeline version 2.10 (9).

The draft genome sequence of LSPQ-04227 is 2,428,218 bp, with an average G+C content of 53.4%. It consists of 10 contigs, ranging in size from 363 to 691,756 bp, with an *N*_50_ of 543,718 bp. A total of 1,772 protein-coding sequences, 51 tRNAs, 5 rRNAs, and 342 pseudo-genes are predicted. The draft genome size of LSPQ-04228 is 2,439,377 bp, with an average G+C content of 53.4%. It consists of 23 contigs, ranging in size from 213 to 410,667 bp, with an N_50_ of 276,589 bp. A total of 1,717 protein-coding sequences, 53 tRNAs, 11 rRNAs, and 424 pseudo-genes are predicted. In strain LSPQ-04227, the *tox* gene is on contig 1 and covers nucleotide positions 202029 to 203711. In strain LSPQ-04228, the *tox* gene is on contig 5, positions 77442 to 79124. Interestingly, whereas in *C. diphtheriae* the *tox* gene is carried by a lysogenic phage, here, an alignment of both *C. ulcerans* tox genes and flanking sequences with *Corynebacterium* phage sequences (10, 11) showed an absence of phage-like sequences, with the exception of an integrase 32 kbp upstream the *tox* gene.

### Nucleotide sequence accession numbers.

These whole-genome shotgun projects have been deposited at the NCBI under the accession numbers JZUS00000000 (strain LSPQ-04227) and JZUT00000000 (strain LSPQ-04228). The versions described in this paper are the first versions, JZUS00000000.1 and JZUT00000000.1, respectively.
